# Comprehensive Profiling of Cell Surface Proteins in Testicular Germ Cell Tumors

**DOI:** 10.1158/2767-9764.CRC-26-0246

**Published:** 2026-07-23

**Authors:** Hedyeh Ebrahimi, Ali Moradi, Regina Barragan-Carrillo, Miguel Zugman, Salvador Jaime-Casas, Koral Shah, Daniela Castro, Benjamin Mercier, Xiaochen Li, Joann Hsu, Peter D. Zang, Charles B. Nguyen, Abhishek Tripathi, Ali Zhumkhawala, Sumanta Kumar Pal, Evita Sadimin, Tanya Dorff, Alex Chehrazi-Raffle

**Affiliations:** 1 Department of Medical Oncology and Experimental Therapeutics, City of Hope Comprehensive Cancer Center, Duarte, California.; 2 Department of Medicine, Beth Israel Deaconess Medical Center, Harvard Medical School, Boston, Massachusetts.; 3 Department of Medical Oncology, Instituto Nacional de Cancerologia, Mexico City, Mexico.; 4 Division of Urology and Urologic Oncology, Department of Surgery, City of Hope Comprehensive Cancer Center, Duarte, California.; 5 Department of Pathology, City of Hope Comprehensive Cancer Center, Duarte, California.

## Abstract

To characterize clinically actionable cell surface proteins in testicular germ cell tumors (GCT), we retrospectively analyzed archival formalin-fixed, paraffin-embedded orchiectomy specimens from 30 patients, including 11 with pure seminoma and 19 with mixed GCTs. Immunohistochemistry was performed for 21 surface antigens using an automated platform. Staining intensity (0 to 3+) and the percentage of positive tumor cells were used to derive H-scores (0–300). Expression was summarized by patient classification and histologic component; high expression was defined as H-score ≥100. Claudin-6 (CLDN6) was diffusely and strongly expressed, with H-score ≥100 in 100% of pure seminomas and 89.5% of mixed GCTs. At the component level, 100% of seminoma and 92.9% of embryonal carcinoma samples reached this threshold. Epidermal growth factor receptor (EGFR) showed high expression in 68.4% of mixed GCTs and 45.5% of pure seminomas, with most enrichment in teratoma components (73.3%, ≥100; mean H-score, 212). TROP2 was markedly upregulated in mixed GCTs (78.9%, ≥100; mean, 194.7), particularly in teratoma components (86.7%, ≥100). Nectin-4 and glycoprotein nonmetastatic melanoma B (GPNMB) were expressed in smaller but notable subsets, including teratoma and seminoma, whereas most remaining antigens, including CD19, DLL3, FOLR1, and BCMA, showed minimal or no expression. These findings demonstrate a distinct surface antigen landscape in testicular GCTs, with CLDN6, EGFR, and TROP2 emerging as leading candidates for further translational evaluation. The enrichment of these targets highlights antigenic heterogeneity within GCTs and supports further validation in appropriate disease settings, including metastatic, posttreatment, and relapsed or refractory disease.

**Significance:**

Relapsed or refractory testicular GCT remains a clinical challenge with limited treatment options. We evaluated cell surface antigens of primary GCTs and identified CLDN6, EGFR, and TROP2 as candidate targets for antigen-directed approaches. These findings provide a rationale for future validation in metastatic and posttreatment disease settings.

## Introduction

Despite the relatively low overall prevalence, testicular cancer is the most common solid tumor in males aged between 15 and 44 years (1). Although multiple cell types within the testis can undergo neoplastic transformation, approximately 90% of testicular cancers originate from germ cells (2, 3). Histologically, germ cell tumors (GCT) are classified into seminomas and nonseminomatous GCTs, which include teratoma, choriocarcinoma, yolk sac tumor, and embryonal carcinoma (2).

GCTs are generally associated with favorable treatment outcomes, with an overall survival rate of approximately 95% in the United States (4). However, the risk of relapse can be as high as 40%, depending on the baseline staging and pathologic features (5, 6). Additionally, certain histologic subtypes, such as mature teratoma and choriocarcinoma, exhibit intrinsic resistance to standard chemotherapy (7–9). Moreover, patients with relapsed or refractory disease often require salvage chemotherapy, which is associated with increased toxicity and variable long-term survival (10, 11). These challenges underscore an urgent need for novel, more targeted treatment strategies.

To improve outcomes, a deeper understanding of the heterogeneous and complex biology of GCT is essential. In this context, surface proteins have emerged as important molecular targets in oncology and offer potential avenues for advancing GCT treatment. These membrane-associated proteins can serve as diagnostic markers, prognostic indicators, or therapeutic targets for strategies such as monoclonal antibodies, antibody–drug conjugates (ADC), and chimeric antigen receptor (CAR) T-cell therapies (12–14).

Despite significant advances in other cancers, comprehensive profiling of surface protein expression across GCT subtypes remains relatively limited. Identifying specific surface proteins that are differentially expressed across histologic subtypes may reveal novel biomarkers related to treatment response and potentially also therapeutic targets. In this study, we performed immunohistochemical profiling of 21 clinically relevant surface antigens across a cohort of GCTs to inform future translational strategies.

## Materials and Methods

### Patient selection

Patients with histologically confirmed testicular GCTs were retrospectively identified through the City of Hope Biospecimen Repository. Eligible cases included individuals who had undergone radical orchiectomy with sufficient archival tumor tissue available for analysis. All patients were ≥18 years of age. Patients with nontesticular GCTs were excluded. Clinical and demographic data were obtained from the electronic medical records. Collected variables included age at diagnosis, race/ethnicity, tumor histology, staging, and metastatic sites at presentation. All patients provided written informed consent for the use of their biospecimens and clinical data. The study was approved by the City of Hope Institutional Review Board (IRB #22202) and was conducted in accordance with the Declaration of Helsinki.

### Tissue processing and immunohistochemistry

Formalin-fixed, paraffin-embedded tumor specimens were obtained from the City of Hope Biospecimen Repository. Hematoxylin and eosin and immunohistochemistry (IHC) slides were prepared at 4 μm thickness and mounted on positively charged glass slides. IHC staining was performed using the Ventana Discovery Ultra automated platform (Ventana Medical Systems, Roche Diagnostics). Slides were processed through deparaffinization, rehydration, inhibition of endogenous peroxidase activity, and antigen retrieval per the manufacturer’s instructions. Primary antibodies were either used as ready-to-use formulations or titrated via serial twofold dilutions based on the manufacturer’s recommendations. Following incubation with primary antibodies, the OptiView DAB IHC Detection Kit (IVD) was used for antigen detection and staining visualization. Slides were counterstained with hematoxylin (Ventana) and coverslipped.

A total of 21 cell surface protein targets were evaluated using validated monoclonal or polyclonal antibodies, including CD19, CD22, CD33, CD37, CD79b, LIV1, epidermal growth factor receptor (EGFR), HER2, PSCA, PSMA, DLL3, FOLR1, claudin-6 (CLDN6), CA6, C-Met, TROP2, BCMA, glycoprotein nonmetastatic melanoma B (GPNMB), TAG-72, Nectin-4, and SALL4 (nuclear staining). Detailed information about the antibody clone, source, and catalog number is provided in Supplementary Table S1.

All slides were scanned using the NanoZoomer S360 Digital Slide Scanner (Hamamatsu) and subsequently analyzed using ImageScope pathology slide viewing software. Staining intensity was graded on a semiquantitative scale as 0 (no staining), 1+ (weak intensity), 2+ (medium intensity), and 3+ (strong intensity). The percentage of tumor cells at each staining intensity was estimated in 5% increments, totaling 100% per specimen. An H-score was calculated using the following formula:

H‐score = [1 × (% of 1+) + 2 × (% of 2+) + 3 × (% of 3+)], yielding a range of 0 to 300.

All cases were evaluated by a board-certified pathologist (E. Sadimin) with expertise in genitourinary pathology, blinded to clinical data.

### Statistical analysis

Descriptive statistics were used to summarize the distribution of H-scores across tumor subtypes (pure seminoma and mixed GCTs) for each of the 21 surface protein targets. H-score distributions were summarized by median [interquartile range (IQR)] and mean ± standard deviation (SD). The proportion of cases with high expression (defined as H-score ≥100) was reported for each marker and histologic component. This threshold was selected as a pragmatic semiquantitative cutoff to facilitate comparison across markers and histologic components. All statistical analyses were conducted using R software (version 4.4.3, R Foundation for Statistical Computing).

## Results

### Patient and sample characteristics

A total of 30 patients with histologically confirmed testicular GCTs were included in this study. Most patients had localized disease at diagnosis, including 19 patients (63.3%) with stage I disease. All specimens analyzed were primary orchiectomy samples; no posttreatment specimens were included. Of these, 11 patients (36.7%) had pure seminoma, and 19 patients (63.3%) had mixed GCTs. The median age at diagnosis for the overall cohort was 29 years (range, 19–81). The majority of patients self-identified as White (*n* = 18; 60%), followed by Asian (*n* = 3; 10%). Ethnicity data showed that 36.7% of the total cohort identified as Hispanic or Latino, 53.3% identified as non-Hispanic or Latino, and 10% had unknown ethnicity. Staging at the time of diagnosis varied across the cohort. Stage 1A was the most common presentation, accounting for 36.7% of patients, followed by stage 1B (20%) and stage 3A (13.3%). A full summary of demographic and clinical characteristics is presented in Table 1.

**Table 1.** Demographic and clinical characteristics of the study cohort (*N* = 30).

Characteristic	Total cohort (*N* = 30)	Pure seminoma (*n* = 11)	Mixed GCTs (*n* = 19)
Age, years			
Median (range)	29 (19–81)	29 (24–81)	25 (19–62)
Race, *n* (%)			
Asian	3 (10%)	2 (18.2%)	1 (5.3%)
American Indian	1 (3.3%)	0 (0%)	1 (5.3%)
White	18 (60%)	6 (54.5%)	12 (63.2%)
Unknown	8 (26.7%)	3 (27.3%)	5 (26.3%)
Ethnicity, *n* (%)			
Hispanic or Latino	11 (36.7%)	3 (27.3%)	8 (42.1%)
Non-Hispanic or Latino	16 (53.3%)	6 (54.5%)	10 (52.6%)
Unknown	3 (10%)	2 (18.2%)	1 (5.3%)
Clinical stage at diagnosis, *n* (%)			
Stage 1A	11 (36.7%)	3 (27.3%)	8 (42.1%)
Stage 1B	6 (20%)	4 (36.4%)	2 (10.5%)
Stage 1S	2 (6.7%)	0 (0%)	2 (10.5%)
Stage 2A	2 (6.7%)	1 (9.1%)	1 (5.3%)
Stage 2B	2 (6.7%)	1 (9.1%)	1 (5.3%)
Stage 2C	1 (3.3%)	1 (9.1%)	0 (0%)
Stage 3A	4 (13.3%)	1 (9.1%)	3 (15.8%)
Stage 3B	0 (0%)	0 (0%)	0 (0%)
Stage 3C	2 (6.7%)	0 (0%)	2 (10.5%)

### CLDN6 expression (membranous staining)

Among patients with pure seminoma (*n* = 11), all cases (100%) exhibited CLDN6 expression with an H-score ≥100. The median H-score was 270 (IQR, 222.5–300), and the mean H-score was 251.4. Strong staining intensity (3+) was observed in 81.8% of pure seminoma cases, with moderate staining (2+) in 63.6% and no cases scoring 0+. Similarly, CLDN6 expression was high in mixed GCTs (*n* = 19), with 89.5% of patients showing an H-score ≥100. The median H-score in this group was 300 (IQR, 295–300), and the mean H-score was 255.8. Strong (3+) staining was present in 89.5% of mixed GCTs, with weak or moderate staining observed less frequently.

At the component level, seminoma components (*n* = 19) had a mean H-score of 246.6, with 100% showing an H-score ≥100. Embryonal carcinoma (*n* = 14) also demonstrated high expression, with 92.9% of samples scoring ≥100 and a mean H-score of 278.6. Yolk sac tumor components (*n* = 11) showed more heterogeneous expression, with 72.7% having an H-score ≥100, with a broad IQR of 55 to 240. In contrast, teratoma components (*n* = 15) had the lowest CLDN6 expression, with only 33.3% reaching an H-score ≥100 and a mean H-score of 66.3. The single choriocarcinoma sample exhibited high CLDN6 expression with an H-score of 300.

### EGFR expression (membranous staining)

Among patients with pure seminoma (*n* = 11), 45.5% exhibited EGFR expression with an H-score ≥100. The median H-score was 90 (IQR, 35–200), and the mean H-score was 111.8. In the mixed GCT group (*n* = 19), 68.4% of patients had an H-score ≥100. The median H-score was 280 (IQR, 90–300), with a mean of 203.4. 3+ staining was observed in 63.2% of cases.

At the component level, seminoma components (*n* = 19) demonstrated an H-score ≥100 in 31.6% of cases, with a mean H-score of 91.1. Embryonal carcinoma (*n* = 14) showed lower EGFR expression overall, with only 7.1% of samples scoring ≥100. Yolk sac tumor components (*n* = 11) had 9.1% reaching an H-score ≥100, with a mean of 59.1. In contrast, teratoma components (*n* = 15) demonstrated higher EGFR expression, with 73.3% showing an H-score ≥100 and a mean H-score of 212. The single choriocarcinoma sample exhibited an H-score of 120.

### TROP2 expression (membranous staining)

In the pure seminoma group (*n* = 11), two patients (18.2%) exhibited TROP2 expression with an H-score ≥100, and the mean H-score was 26.8. However, among patients with mixed GCTs (*n* = 19), 15 (78.9%) had TROP2 H-scores ≥100. The median H-score was 240 (IQR, 112.5–282.5), and the mean H-score was 194.7. Strong staining of 3+ was observed in 78.9% of mixed GCTs.

At the histologic component level, seminoma components (*n* = 19) showed minimal expression, with only 10.5% of samples reaching an H-score ≥100. Within embryonal carcinoma components (*n* = 14), 14.3% reached an H-score of ≥100. Yolk sac tumor components (*n* = 11) had similar findings, with 18.2% showing H-score ≥100. In contrast, teratoma components (*n* = 15) demonstrated the highest TROP2 expression, with 86.7% showing H-score ≥100 and a mean H-score of 224.3. The single choriocarcinoma sample had an H-score of 60.

### Nectin-4 expression with membranous and cytoplasmic staining

Among patients with pure seminoma (*n* = 11), only 1 (9.1%) exhibited an H-score ≥100. The mean H-score in this group was 60.5 (SD 32.6), with most cases showing weak (1+) staining intensity. In the mixed GCT group (*n* = 19), six patients (31.6%) had an H-score ≥100. The median H-score in this cohort was 80 (IQR, 55–120), and the mean was 103.9. Strong (3+) staining was observed in 15.8% of mixed GCTs.

By histologic component, only 10.5% of seminoma components reached an H-score ≥100. Embryonal carcinoma (*n* = 14) and yolk sac tumor components (*n* = 11) had similarly modest expression, with 14.3% and 9.1% reaching an H-score ≥100, respectively. In contrast, teratoma components (*n* = 15) had higher Nectin-4 expression, with 26.7% of samples exceeding an H-score of 100 and a mean H-score of 92.7. The single choriocarcinoma sample showed no Nectin-4 expression.

### HER2 expression (membranous staining)

HER2 expression was absent in all pure seminoma cases (*n* = 11), and no patients exhibited staining above 0+. In contrast, 2 of 19 patients (10.5%) with mixed GCTs demonstrated HER2 expression with an H-score ≥100.

At the component level, seminoma components (*n* = 19) showed no HER2 expression, with all cases scoring 0+. Embryonal carcinoma components (*n* = 14) had a low mean H-score of 11.8, with only 1 sample (7.1%) reaching an H-score ≥100. Yolk sac tumor components (*n* = 11) had similarly low expression levels, with a mean H-score of 11.4 and no samples reaching the 100 threshold for H-score. Teratoma components (*n* = 15) exhibited the highest HER2 expression among components, with a mean H-score of 38, and 1 sample (6.7%) reached an H-score ≥100. The single choriocarcinoma sample had no detectable HER2 expression.

### GPNMB expression (membranous and cytoplasmic staining)

Among patients with pure seminoma cases (*n* = 11), 36.4% (*n* = 4) showed an H-score ≥100 for GPNMB expression. The mean H-score for this group was 108.6, and strong (3+) staining was present in 72.7% of samples. In the mixed GCT group (*n* = 19), the proportion of patients reaching an H-score ≥100 was lower (15.8%). The mean H-score in mixed GCTs was 60.5, but strong staining was observed in 73.7% of cases.

At the component level, seminoma components (*n* = 19) demonstrated relatively high expression, with a mean H-score of 95.5 and 31.6% of cases scoring ≥100. Embryonal carcinoma components (*n* = 14) showed more limited expression, with a mean H-score of 26.4 and no samples reaching the ≥100 threshold. Yolk sac tumor components (*n* = 11) and choriocarcinoma (*n* = 1) exhibited no profound GPNMB expression, with a mean H-score of 3.6 and 0, respectively. Teratoma components (*n* = 15) showed moderate expression, with a mean H-score of 28.7 and 6.7% reaching the ≥100 threshold.

### Surface markers with limited or focal expression

Most of the remaining surface markers in our panel exhibited minimal or absent expression across GCT components. However, a small subset demonstrated modest staining within teratoma components. PSCA expression with cytoplasmic staining reached an H-score ≥100 in 20% of teratoma samples (mean H-score 38.3), whereas it was undetectable or weakly expressed in other components. Similarly, TAG72 with membranous and cytoplasmic staining showed its highest signal in teratoma, with 40% of samples scoring above the H-score threshold of ≥100 and a mean H-score of 87.7, whereas expression in all other histologies remained negligible. c-MET with membranous and cytoplasmic staining was largely absent in all components but reached an H-score ≥100 in 20% of teratomas, with a mean H-score of 62. However, we observed nuclear staining with c-MET in 9 (81.8%) samples with pure seminoma and 11 (57.9%) samples with mixed germ cell tumors. At the component level, 63.1% (*n* = 12) of seminoma components, 57.1% (*n* = 8) of embryonal components, and 72.7% (*n* = 8) of yolk sac components showed nuclear staining with c-MET.

**Figure 1.**
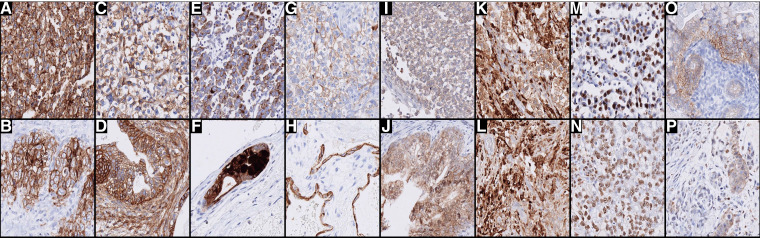
Immunohistochemical staining of selected cell surface markers across testicular GCT subtypes. **A,** CLDN6 (membranous staining) in seminoma, (**B**) CLDN6 (membranous staining) in yolk sac tumor, (**C**) EGFR (membranous staining) in seminoma, (**D**) EGFR (membranous staining) in teratoma, (**E**) TAG72 (membranous and cytoplasmic staining) in seminoma, (**F**) TAG72 (membranous and cytoplasmic staining) in choriocarcinoma, (**G**) TROP2 (membranous staining) in seminoma, (**H**) TROP2 (membranous staining) in teratoma, (**I**) Nectin-4 (membranous and cytoplasmic staining) in seminoma, (**J**) Nectin-4 (membranous and cytoplasmic staining) in teratoma, (**K**) GPNMB (membranous and cytoplasmic staining) in seminoma, (**L**) GPNMB (membranous and cytoplasmic staining) in embryonal carcinoma, (**M**) c-MET (nuclear staining) in seminoma, (**N**) c-MET (nuclear staining) in embryonal carcinoma, (**O**) HER2 (membranous staining) in teratoma, and (**P**) c-MET (membranous and cytoplasmic staining) in choriocarcinoma.

Beyond the aforementioned markers that showed enriched expression in teratoma, the remainder of the panel, including CD19 with membranous staining, CD22 with membranous staining, CD37 with membranous staining, CD79b with membranous staining, CD6 with cytoplasmic staining, DLL3 with membranous and cytoplasmic staining, FOLR1 with membranous staining, LIV1 with membranous and cytoplasmic staining, and PSMA with membranous and cytoplasmic staining, demonstrated minimal to absent surface expression across all GCT components, with no cases reaching the H-score ≥100 threshold. Notably, CD33 with membranous and cytoplasmic staining and BCMA with membranous and cytoplasmic staining exhibited higher H-scores in isolated cases: CD33 surpassed the H-score ≥100 threshold in a seminoma case, and BCMA did so in two samples with embryonal carcinoma although overall expression levels remained limited and inconsistent. Representative IHC expression across different components of GCT is shown in Fig. 1. Detailed expression metrics are provided in Supplementary Table S2.

## Discussion

In this study, we profiled the expression of 21 clinically relevant cell surface proteins across a cohort of testicular GCTs, encompassing both pure seminoma and mixed nonseminomatous histologies. Several markers demonstrated substantial expression across multiple tumor components, with particularly high H-scores and strong staining intensity observed for CLDN6, EGFR, and TROP2. Our findings suggest that cell surface proteins warrant further evaluation as potential therapeutic targets in metastatic and posttreatment specimens.

CLDN6, a tight-junction protein normally silenced in adult tissues, has emerged as a promising oncofetal antigen and therapeutic target due to its restricted expression in embryonal and malignant cells (15, 16). Early-phase clinical studies of CLDN6-targeted strategies, including CAR T cells and ADCs, have shown encouraging activity in CLDN6-positive solid tumors, including GCTs (17–20). In our cohort, CLDN6 demonstrated uniform high expression in embryonal carcinoma (92.9%) and pure seminoma (100%), with 89.5% of mixed GCTs exhibiting H-scores ≥100. These findings align with prior immunohistochemical analyses showing strong and selective CLDN6 expression across GCT subtypes (21). Our findings validate CLDN6 as the most consistently expressed target within a broader panel of clinically actionable surface proteins. Given the high prevalence of CLDN6 in GCTs across multiple studies, prospective CLDN6 screening may have limited utility as a broad selection strategy in testicular GCT.

EGFR demonstrated appreciable expression in our cohort among both patients with mixed GCTs and those with pure seminoma, with particularly high component-level expression in teratoma. These findings mirror earlier studies documenting EGFR overexpression in GCTs using IHC (22, 23). Miyai and colleagues found high *EGFR *expression and amplification in choriocarcinoma components but no mutations in the tyrosine kinase domain, suggesting potential limited utility for mutation-targeted EGFR inhibitors in this disease subtype (24). Consistent with this, Juliachs and colleagues (25) reported that GCT mouse models showed no response to EGFR targeted agents, including gefitinib and cetuximab, whereas partial responses were seen with lapatinib, a dual EGFR/HER2 inhibitor, indicating potential kinase-independent EGFR signaling in testicular tumors. A phase II trial evaluating combined sirolimus and erlotinib (NCT01962896) in patients with relapsed GCT was launched but unfortunately terminated early due to low accrual. Finally, EGFR expression was associated with increased relapse risk in clinical stage I GCTs, suggesting that EGFR may serve as a biomarker in select contexts in addition to its therapeutic implications (26).

TROP2 expression was markedly higher in mixed germ cell tumors than in pure seminoma in our cohort, with the strongest expression observed in teratomas. These findings are consistent with prior studies that also reported TROP2 is widely expressed in GCTs (27–29). TROP2 has emerged as a therapeutic candidate in various malignancies, with TROP2-targeted ADCs such as sacituzumab govitecan being clinically approved in other cancers (30). Importantly, a recent preclinical study by Sperber showed strong cytotoxicity of sacituzumab govitecan in cisplatin-resistant GCT cell lines. Similarly, treatment of GCT cell line–derived xenograft models and patient-derived xenograft models with sacituzumab govitecan led to reduced tumor volume (31). This study also reported a clinical case in which a patient with refractory testicular GCT achieved tumor marker normalization, improved performance status, and tumor burden control following sacituzumab govitecan therapy (31).

However, the enrichment of EGFR and TROP2 expression in teratoma components should be interpreted cautiously. In our cohort, most tumors with teratoma components were mature, which are generally managed surgically and are not typically considered an indication for systemic targeted therapy. Therefore, our findings should not be interpreted as supporting routine EGFR- or TROP2-directed therapy for mature teratoma. Rather, these results highlight antigenic heterogeneity within mixed GCTs and support further evaluation of these targets in selected clinical settings where systemic approaches may be relevant, such as growing teratoma syndrome or posttreatment residual teratoma.

Although Nectin-4 was not as broadly expressed in our cohort as other surface targets such as CLDN6, EGFR, or TROP2, a substantial subset of testicular GCTs nevertheless demonstrated appreciable expression. H-scores ≥100 were observed in 31.6% of patients with mixed GCTs and 9.1% of patients with pure seminoma. A recent immunohistochemical study of 46 testicular GCT samples reported variable Nectin-4 expression, with stronger positivity observed in choriocarcinoma components (32). Therapeutically, enfortumab vedotin, a Nectin-4 directed ADC, has demonstrated a survival benefit and received FDA approval for advanced urothelial carcinoma (33). Building on its success in urothelial malignancies, a phase II trial of enfortumab vedotin with or without pembrolizumab targeting patients with refractory testicular cancer is underway (NCT06041503).

In our cohort, HER2 expression was absent in pure seminoma and minimal across most nonseminomatous components. These findings are consistent with prior studies that demonstrated weak cell surface HER2 expression in testicular GCTs (34–36). These data indicate that HER2 is not widely expressed in GCTs and should not be pursued as a potential therapeutic target.

GPNMB expression was observed in a subset of testicular GCTs in our study, with strong staining seen in seminoma and teratoma components. To date, no formal studies have specifically evaluated GPNMB expression or therapeutic targeting in GCTs. GPNMB has previously been explored as a drug target in other malignancies using glembatumumab vedotin, a GPNMB-directed ADC; however, the development was halted following the negative results of the phase IIb METRIC trial in triple-negative breast cancer (37). Given the subset expression observed in our cohort, further exploration of GPNMB as a potential therapeutic target in GCTs, particularly in refractory cases or subtypes with limited treatment options.

Although the majority of the remaining 14 evaluated markers, such as CD19, CD22, CD33, CD79b, CD6, CD37, LIV1, DLL3, PSMA, FOLR1, and BCMA, demonstrated minimal or no expression across testicular GCT components in our cohort, their assessment remains a valuable aspect of this study, as a few cases in our cohort showed strong expression of these markers. These findings suggest that although most of these targets lack appreciable surface expression in testicular GCTs, a few may warrant further exploration in select histologies or subpopulations. Several of these targets have well-established therapeutic relevance in other malignancies, particularly in the context of ADC, including CD79b in B-cell lymphomas, DLL3 in small cell lung cancer, and LIV1 in breast cancer (38–40). Despite the limited expression observed, systematically ruling out these antigens helps refine the pool of actionable targets in testicular cancer and underscores the comprehensive nature of our antigen discovery approach. Continued exploration of surface targets across diverse histologies and treatment-resistant disease states remains critical for advancing GCT-directed therapeutics.

Our study has several limitations worth noting. First, our cohort largely consisted of patients with localized disease, and all analyses were performed on archived primary orchiectomy specimens. Although this approach reflects a feasible tissue source for immunohistochemical profiling in testicular GCTs, it may not fully capture the antigenic landscape of metastatic, posttreatment, or relapsed/refractory disease, as tumor biology may evolve under the selective pressure of chemotherapy. Therefore, the applicability of these findings to patients with heavily pretreated or refractory GCT requires further validation in metastatic and posttreatment specimens. In addition, archived tissue may be subject to antigen alterations or loss over time, as well as variability in interpretation. Although H-scores were consistently calculated, IHC remains a semiquantitative technique. Our analysis was also limited to protein expression by IHC and did not include genomic or transcriptomic profiling. Furthermore, our sample size was substantial for a rare tumor type, but it may still limit generalizability. This is especially true for less common histologic subtypes in our cohort, such as choriocarcinoma. Finally, we surveyed a broad panel of 21 cell surface markers with known therapeutic relevance in other malignancies. However, emerging or GCT-specific antigens that have not yet been clinically characterized may have been missed.

In conclusion, this comparative assessment of clinically actionable cell surface proteins of testicular GCTs identified CLDN6, EGFR, and TROP2 as the most relevant targets for further translational evaluation. CLDN6 was the most consistently expressed marker, supporting its continued therapeutic development in GCTs. Future studies incorporating functional validation, larger multicenter cohorts, metastatic and posttreatment specimens, genomic and transcriptomic profiling, and correlation with clinical outcomes will be crucial for defining the clinical utility of these targets and refining target selection in testicular GCTs.

## Supplementary Material

Supplementary Table 1 Antibodies used for immunohistochemistry, including clone, catalog number, and vendor.

Supplementary Table 2 Immunohistochemical expression for 21 cell surface markers across testicular germ cell tumor subtypes and histologic components.

## Data Availability

The data generated in this study are available from the corresponding author upon reasonable request. Data are not publicly available due to Institutional Review Board and patient privacy restrictions.
