# Supplementary material for: Comprehensive Profiling of Cell Surface Proteins in Testicular Germ Cell Tumors
Source: Cancer Res Commun. 2026 Jul 23;6(7):1762–8. doi: 10.1158/2767-9764.CRC-26-0246 (PMC13392819; doi:10.1158/2767-9764.CRC-26-0246)
Supplement: Supplementary Table 1 — Antibodies used for immunohistochemistry, including clone, catalog number, and vendor. [file crc-26-0246_supplementary_table_1_suppst1.docx]

**Supplementary Table 1. Antibodies used for immunohistochemistry, including clone, catalog number, and vendor.**

| **Target antigen** | **Clone** | **Catalog number** | **Antibody type** | **Vendor** |
| --- | --- | --- | --- | --- |
| **CD19** | EP169 | 119R-14 | Rabbit monoclonal | Cell Marque |
| **CD79B** | D7V2F | 96024 | Rabbit monoclonal | Cell Signaling |
| **HER2** | 4B5 | 790-2991 | Rabbit monoclonal | Ventana |
| **Nectin-4** | Poly | AF2659 | Rabbit polyclonal | R&D System |
| **TROP-2** | EPR20043 | AB214488 | Rabbit monoclonal | ABCAM |
| **BCMA** | D-6 | SC-390147 | Mouse monoclonal | SCBT |
| **EGFR** | EP22 | AC-0025 | Rabbit monoclonal | Cell Marque |
| **PSCA** | 5C2 | H00008000-M03 | Mouse monoclonal | ABNOVA |
| **TAG-72** | CC49 | NBP2-31383 | Mouse monoclonal | NOVUS |
| **CLDN6** | E7U2O | 18932 | Rabbit monoclonal | Cell Signaling |
| **PSMA** | EP192 | 760-6077 | Rabbit monoclonal | Ventana |
| **CD22** | E7L6Z | 98035 | Rabbit monoclonal | Cell Signaling |
| **CD33** | PWS44 | 133M-15 | Mouse monoclonal | Cell Marque |
| **DLL3** | E3J5R | 71804 | Rabbit monoclonal | Cell Signaling |
| **FOLR1** | BN3.2 | 37283 | Mouse monoclonal | Cell Signaling |
| **CD37** | EPR25397-149 | AB300400 | Rabbit monoclonal | ABCAM |
| **CD6** | EPR13954(2) | AB210406 | Rabbit monoclonal | ABCAM |
| **GPNMB** | E4D7P | 38313 | Rabbit monoclonal | Cell Signaling |
| **C-Met** | SP44 | 790-4430 | Rabbit monoclonal | Ventana |
| **ZIP-6 (LIV1)** | SP300 | AB227696 | Rabbit monoclonal | ABCAM |
| **SALL4** | 6E3 | 385M-15 | Mouse monoclonal | Cell Marque |
