# Supplementary material for: Comprehensive Profiling of Cell Surface Proteins in Testicular Germ Cell Tumors
Source: Cancer Res Commun. 2026 Jul 23;6(7):1762–8. doi: 10.1158/2767-9764.CRC-26-0246 (PMC13392819; doi:10.1158/2767-9764.CRC-26-0246)
Supplement: Supplementary Table 2 — Immunohistochemical expression for 21 cell surface markers across testicular germ cell tumor subtypes and histologic components. [file crc-26-0246_supplementary_table_2_suppst2.docx]

**Supplementary Table 2. Immunohistochemical expression for 21 cell surface markers across testicular germ cell tumor subtypes and histologic components.**

| Marker | Histology | | Number of samples | H-score Median | H-score Q1 | H-score Q3 | H-score min | H-score max | H-score  Mean | H-score  SD | H-score ≥100 | |
| --- | --- | --- | --- | --- | --- | --- | --- | --- | --- | --- | --- | --- |
|  |  |  |  |  |  |  |  |  |  |  | Number | % |
| SALL4 | Tumor subtype | Pure Seminoma | 11 | 300 | 300 | 300 | 300 | 300 | 300.0 | 0.0 | 11 | 100.0 |
|  |  | Mixed Germ Cell Tumor | 19 | 300 | 300 | 300 | 0 | 300 | 268.4 | 94.6 | 17 | 89.5 |
|  | Component | Seminoma | 19 | 300 | 300 | 300 | 200 | 300 | 294.7 | 22.9 | 19 | 100.0 |
|  |  | Embryonal | 14 | 300 | 300 | 300 | 40 | 300 | 281.4 | 69.5 | 13 | 92.9 |
|  |  | Yolk Sac | 11 | 300 | 280 | 300 | 90 | 300 | 263.6 | 73.2 | 10 | 90.9 |
|  |  | Teratoma | 15 | 0 | 0 | 0 | 0 | 30 | 4.0 | 9.1 | 0 | 0.0 |
|  |  | Choriocarcinoma | 1 | 300 | 300 | 300 | 300 | 300 | 300.0 | NA | 1 | 100.0 |
|  |  | GCNIS | 24 | 300 | 300 | 300 | 200 | 300 | 295.8 | 20.4 | 24 | 100.0 |
| EGFR | Tumor subtype | Pure Seminoma | 11 | 90 | 35 | 200 | 0 | 260 | 111.8 | 92.3 | 5 | 45.5 |
|  |  | Mixed Germ Cell Tumor | 19 | 280 | 90 | 300 | 40 | 300 | 203.4 | 104.4 | 13 | 68.4 |
|  | Component | Seminoma | 19 | 80 | 35 | 120 | 0 | 260 | 91.1 | 76.8 | 6 | 31.6 |
|  |  | Embryonal | 14 | 0 | 0 | 30 | 0 | 100 | 24.3 | 36.6 | 1 | 7.1 |
|  |  | Yolk Sac | 11 | 30 | 0 | 75 | 0 | 300 | 59.1 | 86.8 | 1 | 9.1 |
|  |  | Teratoma | 15 | 290 | 125 | 300 | 0 | 300 | 212.0 | 115.5 | 11 | 73.3 |
|  |  | Choriocarcinoma | 1 | 120 | 120 | 120 | 120 | 120 | 120.0 | NA | 1 | 100.0 |
|  |  | GCNIS | 24 | 0 | 0 | 0 | 0 | 0 | 0.0 | 0.0 | 0 | 0.0 |
| CD79b | Tumor subtype | Pure Seminoma | 11 | 0 | 0 | 0 | 0 | 0 | 0.0 | 0.0 | 0 | 0.0 |
|  |  | Mixed Germ Cell Tumor | 19 | 0 | 0 | 0 | 0 | 0 | 0.0 | 0.0 | 0 | 0.0 |
|  | Component | Seminoma | 19 | 0 | 0 | 0 | 0 | 0 | 0.0 | 0.0 | 0 | 0.0 |
|  |  | Embryonal | 14 | 0 | 0 | 0 | 0 | 0 | 0.0 | 0.0 | 0 | 0.0 |
|  |  | Yolk Sac | 11 | 0 | 0 | 0 | 0 | 0 | 0.0 | 0.0 | 0 | 0.0 |
|  |  | Teratoma | 15 | 0 | 0 | 0 | 0 | 0 | 0.0 | 0.0 | 0 | 0.0 |
|  |  | Choriocarcinoma | 1 | 0 | 0 | 0 | 0 | 0 | 0.0 | NA | 0 | 0.0 |
|  |  | GCNIS | 24 | 0 | 0 | 0 | 0 | 0 | 0.0 | 0.0 | 0 | 0.0 |
| PSMA | Tumor subtype | Pure Seminoma | 11 | 0 | 0 | 0 | 0 | 10 | 0.9 | 3.0 | 0 | 0.0 |
|  |  | Mixed Germ Cell Tumor | 19 | 10 | 0 | 25 | 0 | 80 | 18.4 | 24.3 | 0 | 0.0 |
|  | Component | Seminoma | 19 | 0 | 0 | 0 | 0 | 10 | 0.5 | 2.3 | 0 | 0.0 |
|  |  | Embryonal | 14 | 0 | 0 | 0 | 0 | 80 | 5.7 | 21.4 | 0 | 0.0 |
|  |  | Yolk Sac | 11 | 10 | 0 | 20 | 0 | 60 | 16.4 | 21.1 | 0 | 0.0 |
|  |  | Teratoma | 15 | 0 | 0 | 2.5 | 0 | 50 | 6.3 | 13.9 | 0 | 0.0 |
|  |  | Choriocarcinoma | 1 | 0 | 0 | 0 | 0 | 0 | 0.0 | NA | 0 | 0.0 |
|  |  | GCNIS | 24 | 0 | 0 | 0 | 0 | 0 | 0.0 | 0.0 | 0 | 0.0 |
| CD19 | Tumor subtype | Pure Seminoma | 11 | 0 | 0 | 0 | 0 | 0 | 0.0 | 0.0 | 0 | 0.0 |
|  |  | Mixed Germ Cell Tumor | 19 | 0 | 0 | 0 | 0 | 0 | 0.0 | 0.0 | 0 | 0.0 |
|  | Component | Seminoma | 19 | 0 | 0 | 0 | 0 | 0 | 0.0 | 0.0 | 0 | 0.0 |
|  |  | Embryonal | 14 | 0 | 0 | 0 | 0 | 0 | 0.0 | 0.0 | 0 | 0.0 |
|  |  | Yolk Sac | 11 | 0 | 0 | 0 | 0 | 0 | 0.0 | 0.0 | 0 | 0.0 |
|  |  | Teratoma | 15 | 0 | 0 | 0 | 0 | 0 | 0.0 | 0.0 | 0 | 0.0 |
|  |  | Choriocarcinoma | 1 | 0 | 0 | 0 | 0 | 0 | 0.0 | NA | 0 | 0.0 |
|  |  | GCNIS | 24 | 0 | 0 | 0 | 0 | 0 | 0.0 | 0.0 | 0 | 0.0 |
| CD6 | Tumor subtype | Pure Seminoma | 11 | 0 | 0 | 0 | 0 | 0 | 0.0 | 0.0 | 0 | 0.0 |
|  |  | Mixed Germ Cell Tumor | 19 | 0 | 0 | 0 | 0 | 12 | 1.2 | 3.5 | 0 | 0.0 |
|  | Component | Seminoma | 19 | 0 | 0 | 0 | 0 | 0 | 0.0 | 0.0 | 0 | 0.0 |
|  |  | Embryonal | 14 | 0 | 0 | 0 | 0 | 0 | 0.0 | 0.0 | 0 | 0.0 |
|  |  | Yolk Sac | 11 | 0 | 0 | 0 | 0 | 0 | 0.0 | 0.0 | 0 | 0.0 |
|  |  | Teratoma | 15 | 0 | 0 | 0 | 0 | 12 | 1.5 | 3.9 | 0 | 0.0 |
|  |  | Choriocarcinoma | 1 | 0 | 0 | 0 | 0 | 0 | 0.0 | NA | 0 | 0.0 |
|  |  | GCNIS | 24 | 0 | 0 | 0 | 0 | 0 | 0.0 | 0.0 | 0 | 0.0 |
| GPNMB | Tumor subtype | Pure Seminoma | 11 | 70 | 40 | 162.5 | 0 | 285 | 108.6 | 103.6 | 4 | 36.4 |
|  |  | Mixed Germ Cell Tumor | 19 | 50 | 25 | 70 | 0 | 260 | 60.5 | 62.7 | 3 | 15.8 |
|  | Component | Seminoma | 19 | 70 | 25 | 130 | 0 | 285 | 95.5 | 96.2 | 6 | 31.6 |
|  |  | Embryonal | 14 | 15 | 0 | 50 | 0 | 90 | 26.4 | 28.9 | 0 | 0.0 |
|  |  | Yolk Sac | 11 | 0 | 0 | 0 | 0 | 20 | 3.6 | 8.1 | 0 | 0.0 |
|  |  | Teratoma | 15 | 30 | 0 | 30 | 0 | 140 | 28.7 | 36.7 | 1 | 6.7 |
|  |  | Choriocarcinoma | 1 | 0 | 0 | 0 | 0 | 0 | 0.0 | NA | 0 | 0.0 |
|  |  | GCNIS | 24 | 0 | 0 | 0 | 0 | 0 | 0.0 | 0.0 | 0 | 0.0 |
| LIV1 | Tumor subtype | Pure Seminoma | 11 | 0 | 0 | 0 | 0 | 0 | 0.0 | 0.0 | 0 | 0.0 |
|  |  | Mixed Germ Cell Tumor | 19 | 0 | 0 | 0 | 0 | 0 | 0.0 | 0.0 | 0 | 0.0 |
|  | Component | Seminoma | 19 | 0 | 0 | 0 | 0 | 0 | 0.0 | 0.0 | 0 | 0.0 |
|  |  | Embryonal | 14 | 0 | 0 | 0 | 0 | 0 | 0.0 | 0.0 | 0 | 0.0 |
|  |  | Yolk Sac | 11 | 0 | 0 | 0 | 0 | 0 | 0.0 | 0.0 | 0 | 0.0 |
|  |  | Teratoma | 15 | 0 | 0 | 0 | 0 | 0 | 0.0 | 0.0 | 0 | 0.0 |
|  |  | Choriocarcinoma | 1 | 0 | 0 | 0 | 0 | 0 | 0.0 | NA | 0 | 0.0 |
|  |  | GCNIS | 24 | 0 | 0 | 0 | 0 | 0 | 0.0 | 0.0 | 0 | 0.0 |
| DLL3 | Tumor subtype | Pure Seminoma | 11 | 0 | 0 | 0 | 0 | 30 | 2.7 | 9.0 | 0 | 0.0 |
|  |  | Mixed Germ Cell Tumor | 19 | 0 | 0 | 5 | 0 | 90 | 12.7 | 27.4 | 0 | 0.0 |
|  | Component | Seminoma | 19 | 0 | 0 | 0 | 0 | 30 | 1.6 | 6.9 | 0 | 0.0 |
|  |  | Embryonal | 14 | 1 | 0 | 16.25 | 0 | 90 | 17.3 | 30.8 | 0 | 0.0 |
|  |  | Yolk Sac | 11 | 0 | 0 | 0 | 0 | 0 | 0.0 | 0.0 | 0 | 0.0 |
|  |  | Teratoma | 15 | 0 | 0 | 0 | 0 | 40 | 2.7 | 10.3 | 0 | 0.0 |
|  |  | Choriocarcinoma | 1 | 0 | 0 | 0 | 0 | 0 | 0.0 | NA | 0 | 0.0 |
|  |  | GCNIS | 24 | 0 | 0 | 0 | 0 | 0 | 0.0 | 0.0 | 0 | 0.0 |
| CD37 | Tumor subtype | Pure Seminoma | 11 | 0 | 0 | 0 | 0 | 10 | 1.4 | 3.2 | 0 | 0.0 |
|  |  | Mixed Germ Cell Tumor | 19 | 0 | 0 | 0 | 0 | 30 | 1.8 | 6.9 | 0 | 0.0 |
|  | Component | Seminoma | 19 | 0 | 0 | 0 | 0 | 10 | 1.1 | 2.7 | 0 | 0.0 |
|  |  | Embryonal | 14 | 0 | 0 | 0 | 0 | 10 | 1.1 | 2.9 | 0 | 0.0 |
|  |  | Yolk Sac | 11 | 0 | 0 | 0 | 0 | 0 | 0.0 | 0.0 | 0 | 0.0 |
|  |  | Teratoma | 15 | 0 | 0 | 0 | 0 | 30 | 2.0 | 7.7 | 0 | 0.0 |
|  |  | Choriocarcinoma | 1 | 0 | 0 | 0 | 0 | 0 | 0.0 | NA | 0 | 0.0 |
|  |  | GCNIS | 24 | 0 | 0 | 0 | 0 | 0 | 0.0 | 0.0 | 0 | 0.0 |
| HER2 | Tumor subtype | Pure Seminoma | 11 | 0 | 0 | 0 | 0 | 0 | 0.0 | 0.0 | 0 | 0.0 |
|  |  | Mixed Germ Cell Tumor | 19 | 20 | 0 | 70 | 0 | 120 | 36.3 | 41.5 | 2 | 10.5 |
|  | Component | Seminoma | 19 | 0 | 0 | 0 | 0 | 0 | 0.0 | 0.0 | 0 | 0.0 |
|  |  | Embryonal | 14 | 0 | 0 | 3.75 | 0 | 110 | 11.8 | 30.2 | 1 | 7.1 |
|  |  | Yolk Sac | 11 | 0 | 0 | 20 | 0 | 60 | 11.4 | 18.5 | 0 | 0.0 |
|  |  | Teratoma | 15 | 40 | 0 | 70 | 0 | 120 | 38.0 | 40.6 | 1 | 6.7 |
|  |  | Choriocarcinoma | 1 | 0 | 0 | 0 | 0 | 0 | 0.0 | NA | 0 | 0.0 |
|  |  | GCNIS | 24 | 0 | 0 | 0 | 0 | 0 | 0.0 | 0.0 | 0 | 0.0 |
| TROP2 | Tumor subtype | Pure Seminoma | 11 | 0 | 0 | 0 | 0 | 175 | 26.8 | 60.9 | 2 | 18.2 |
|  |  | Mixed Germ Cell Tumor | 19 | 240 | 112.5 | 282.5 | 0 | 300 | 194.7 | 102.8 | 15 | 78.9 |
|  | Component | Seminoma | 19 | 0 | 0 | 0 | 0 | 175 | 15.5 | 47.4 | 2 | 10.5 |
|  |  | Embryonal | 14 | 15 | 0 | 75 | 0 | 130 | 37.5 | 47.2 | 2 | 14.3 |
|  |  | Yolk Sac | 11 | 0 | 0 | 67.5 | 0 | 170 | 38.6 | 56.8 | 2 | 18.2 |
|  |  | Teratoma | 15 | 250 | 210 | 285 | 0 | 300 | 224.3 | 91.6 | 13 | 86.7 |
|  |  | Choriocarcinoma | 1 | 60 | 60 | 60 | 60 | 60 | 60.0 | NA | 0 | 0.0 |
|  |  | GCNIS | 24 | 0 | 0 | 0 | 0 | 0 | 0.0 | 0.0 | 0 | 0.0 |
| BCMA | Tumor subtype | Pure Seminoma | 11 | 5 | 2.5 | 10 | 0 | 20 | 7.3 | 7.2 | 0 | 0.0 |
|  |  | Mixed Germ Cell Tumor | 19 | 10 | 0 | 30 | 0 | 220 | 31.3 | 54.1 | 2 | 10.5 |
|  | Component | Seminoma | 19 | 5 | 0 | 10 | 0 | 80 | 10.8 | 18.1 | 0 | 0.0 |
|  |  | Embryonal | 14 | 0 | 0 | 17.5 | 0 | 140 | 25.0 | 47.5 | 2 | 14.3 |
|  |  | Yolk Sac | 11 | 0 | 0 | 0 | 0 | 30 | 2.7 | 9.0 | 0 | 0.0 |
|  |  | Teratoma | 15 | 10 | 0 | 20 | 0 | 220 | 24.7 | 55.4 | 1 | 6.7 |
|  |  | Choriocarcinoma | 1 | 0 | 0 | 0 | 0 | 0 | 0.0 | NA | 0 | 0.0 |
|  |  | GCNIS | 24 | 0 | 0 | 0 | 0 | 0 | 0.0 | 0.0 | 0 | 0.0 |
| CD22 | Tumor subtype | Pure Seminoma | 11 | 0 | 0 | 0 | 0 | 10 | 0.9 | 3.0 | 0 | 0.0 |
|  |  | Mixed Germ Cell Tumor | 19 | 0 | 0 | 0 | 0 | 30 | 1.6 | 6.9 | 0 | 0.0 |
|  | Component | Seminoma | 19 | 0 | 0 | 0 | 0 | 30 | 2.1 | 7.1 | 0 | 0.0 |
|  |  | Embryonal | 14 | 0 | 0 | 0 | 0 | 0 | 0.0 | 0.0 | 0 | 0.0 |
|  |  | Yolk Sac | 11 | 0 | 0 | 0 | 0 | 0 | 0.0 | 0.0 | 0 | 0.0 |
|  |  | Teratoma | 15 | 0 | 0 | 0 | 0 | 0 | 0.0 | 0.0 | 0 | 0.0 |
|  |  | Choriocarcinoma | 1 | 0 | 0 | 0 | 0 | 0 | 0.0 | NA | 0 | 0.0 |
|  |  | GCNIS | 24 | 0 | 0 | 0 | 0 | 0 | 0.0 | 0.0 | 0 | 0.0 |
| PSCA | Tumor subtype | Pure Seminoma | 11 | 0 | 0 | 0 | 0 | 0 | 0.0 | 0.0 | 0 | 0.0 |
|  |  | Mixed Germ Cell Tumor | 19 | 0 | 0 | 45 | 0 | 200 | 32.9 | 56.4 | 3 | 15.8 |
|  | Component | Seminoma | 19 | 0 | 0 | 0 | 0 | 0 | 0.0 | 0.0 | 0 | 0.0 |
|  |  | Embryonal | 14 | 0 | 0 | 0 | 0 | 5 | 0.4 | 1.3 | 0 | 0.0 |
|  |  | Yolk Sac | 11 | 0 | 0 | 0 | 0 | 45 | 4.1 | 13.6 | 0 | 0.0 |
|  |  | Teratoma | 15 | 0 | 0 | 62.5 | 0 | 200 | 38.3 | 61.9 | 3 | 20.0 |
|  |  | Choriocarcinoma | 1 | 0 | 0 | 0 | 0 | 0 | 0.0 | NA | 0 | 0.0 |
|  |  | GCNIS | 24 | 0 | 0 | 0 | 0 | 0 | 0.0 | 0.0 | 0 | 0.0 |
| CD33 | Tumor subtype | Pure Seminoma | 11 | 5 | 2.5 | 10 | 0 | 90 | 14.1 | 26.2 | 0 | 0.0 |
|  |  | Mixed Germ Cell Tumor | 19 | 0 | 0 | 0 | 0 | 110 | 6.3 | 25.2 | 1 | 5.3 |
|  | Component | Seminoma | 19 | 5 | 0 | 7.5 | 0 | 110 | 14.2 | 31.0 | 1 | 5.3 |
|  |  | Embryonal | 14 | 0 | 0 | 0 | 0 | 5 | 0.4 | 1.3 | 0 | 0.0 |
|  |  | Yolk Sac | 11 | 0 | 0 | 0 | 0 | 0 | 0.0 | 0.0 | 0 | 0.0 |
|  |  | Teratoma | 15 | 0 | 0 | 0 | 0 | 0 | 0.0 | 0.0 | 0 | 0.0 |
|  |  | Choriocarcinoma | 1 | 0 | 0 | 0 | 0 | 0 | 0.0 | NA | 0 | 0.0 |
|  |  | GCNIS | 24 | 0 | 0 | 0 | 0 | 0 | 0.0 | 0.0 | 0 | 0.0 |
| FOLR1 | Tumor subtype | Pure Seminoma | 11 | 0 | 0 | 0 | 0 | 0 | 0.0 | 0.0 | 0 | 0.0 |
|  |  | Mixed Germ Cell Tumor | 19 | 0 | 0 | 7.5 | 0 | 90 | 11.1 | 24.2 | 0 | 0.0 |
|  | Component | Seminoma | 19 | 0 | 0 | 0 | 0 | 0 | 0.0 | 0.0 | 0 | 0.0 |
|  |  | Embryonal | 14 | 0 | 0 | 0 | 0 | 0 | 0.0 | 0.0 | 0 | 0.0 |
|  |  | Yolk Sac | 11 | 0 | 0 | 0 | 0 | 90 | 8.2 | 27.1 | 0 | 0.0 |
|  |  | Teratoma | 15 | 0 | 0 | 15 | 0 | 70 | 12.7 | 22.7 | 0 | 0.0 |
|  |  | Choriocarcinoma | 1 | 0 | 0 | 0 | 0 | 0 | 0.0 | NA | 0 | 0.0 |
|  |  | GCNIS | 24 | 0 | 0 | 0 | 0 | 0 | 0.0 | 0.0 | 0 | 0.0 |
| CLDN6 | Tumor subtype | Pure Seminoma | 11 | 270 | 222.5 | 300 | 130 | 300 | 251.4 | 54.6 | 11 | 100.0 |
|  |  | Mixed Germ Cell Tumor | 19 | 300 | 295 | 300 | 0 | 300 | 255.8 | 97.4 | 17 | 89.5 |
|  | Component | Seminoma | 19 | 270 | 200 | 300 | 130 | 300 | 246.6 | 56.9 | 19 | 100.0 |
|  |  | Embryonal | 14 | 300 | 300 | 300 | 0 | 300 | 278.6 | 80.2 | 13 | 92.9 |
|  |  | Yolk Sac | 11 | 200 | 55 | 240 | 0 | 300 | 155.5 | 117.3 | 8 | 72.7 |
|  |  | Teratoma | 15 | 30 | 0 | 137.5 | 0 | 270 | 66.3 | 84.1 | 5 | 33.3 |
|  |  | Choriocarcinoma | 1 | 300 | 300 | 300 | 300 | 300 | 300.0 | NA | 1 | 100.0 |
|  |  | GCNIS | 24 | 200 | 200 | 200 | 0 | 300 | 187.1 | 66.1 | 23 | 95.8 |
| TAG72 | Tumor subtype | Pure Seminoma | 11 | 15 | 0 | 37.5 | 0 | 90 | 27.3 | 34.0 | 0 | 0.0 |
|  |  | Mixed Germ Cell Tumor | 19 | 55 | 0 | 120 | 0 | 240 | 76.6 | 84.5 | 6 | 31.6 |
|  | Component | Seminoma | 19 | 0 | 0 | 27.5 | 0 | 90 | 20.0 | 32.1 | 0 | 0.0 |
|  |  | Embryonal | 14 | 0 | 0 | 0 | 0 | 55 | 5.4 | 15.2 | 0 | 0.0 |
|  |  | Yolk Sac | 11 | 0 | 0 | 0 | 0 | 50 | 5.5 | 15.1 | 0 | 0.0 |
|  |  | Teratoma | 15 | 55 | 30 | 120 | 0 | 240 | 87.7 | 89.0 | 6 | 40.0 |
|  |  | Choriocarcinoma | 1 | 90 | 90 | 90 | 90 | 90 | 90.0 | NA | 0 | 0.0 |
|  |  | GCNIS | 24 | 0 | 0 | 0 | 0 | 0 | 0.0 | 0.0 | 0 | 0.0 |
| Nectin4 | Tumor subtype | Pure Seminoma | 11 | 60 | 42.5 | 80 | 0 | 120 | 60.5 | 32.6 | 1 | 9.1 |
|  |  | Mixed Germ Cell Tumor | 19 | 80 | 55 | 120 | 0 | 285 | 103.9 | 80.1 | 6 | 31.6 |
|  | Component | Seminoma | 19 | 60 | 35 | 80 | 0 | 190 | 60.3 | 44.6 | 2 | 10.5 |
|  |  | Embryonal | 14 | 65 | 20 | 77.5 | 0 | 200 | 63.6 | 61.6 | 2 | 14.3 |
|  |  | Yolk Sac | 11 | 0 | 0 | 55 | 0 | 120 | 29.5 | 47.2 | 1 | 9.1 |
|  |  | Teratoma | 15 | 70 | 25 | 107.5 | 0 | 285 | 92.7 | 94.4 | 4 | 26.7 |
|  |  | Choriocarcinoma | 1 | 0 | 0 | 0 | 0 | 0 | 0.0 | NA | 0 | 0.0 |
|  |  | GCNIS | 24 | 0 | 0 | 0 | 0 | 0 | 0.0 | 0.0 | 0 | 0.0 |
| c-Met | Tumor subtype | Pure Seminoma | 11 | 0 | 0 | 0 | 0 | 0 | 0.0 | 0.0 | 0 | 0.0 |
|  |  | Mixed Germ Cell Tumor | 19 | 0 | 0 | 75 | 0 | 290 | 48.9 | 78.5 | 3 | 15.8 |
|  | Component | Seminoma | 19 | 0 | 0 | 0 | 0 | 0 | 0.0 | 0.0 | 0 | 0.0 |
|  |  | Embryonal | 14 | 0 | 0 | 0 | 0 | 0 | 0.0 | 0.0 | 0 | 0.0 |
|  |  | Yolk Sac | 11 | 0 | 0 | 0 | 0 | 0 | 0.0 | 0.0 | 0 | 0.0 |
|  |  | Teratoma | 15 | 40 | 0 | 80 | 0 | 290 | 62.0 | 84.0 | 3 | 20.0 |
|  |  | Choriocarcinoma | 1 | 60 | 60 | 60 | 60 | 60 | 60.0 | NA | 0 | 0.0 |
|  |  | GCNIS | 24 | 0 | 0 | 0 | 0 | 0 | 0.0 | 0.0 | 0 | 0.0 |
